# Concurrent host-pathogen gene expression in the lungs of pigs challenged with *Actinobacillus pleuropneumoniae*

**DOI:** 10.1186/s12864-015-1557-6

**Published:** 2015-05-28

**Authors:** Louise Brogaard, Kirstine Klitgaard, Peter MH Heegaard, Mette Sif Hansen, Tim Kåre Jensen, Kerstin Skovgaard

**Affiliations:** Innate Immunology Group, Section of Immunology and Vaccinology, National Veterinary Institute, Technical University of Denmark, Bülowsvej 27, 1870 Frederiksberg C, Denmark; Section of Bacteriology, Pathology and Parasitology, National Veterinary Institute, Technical University of Denmark, Bülowsvej 27, 1870 Frederiksberg C, Denmark

**Keywords:** High-throughput RT-qPCR, Transcriptional analysis, Host-pathogen interactions, Innate immunity, *Actinobacillus pleuropneumoniae*, Respiratory infection, Laser capture microdissection

## Abstract

**Background:**

*Actinobacillus pleuropneumoniae* causes pleuropneumonia in pigs, a disease which is associated with high morbidity and mortality, as well as impaired animal welfare. To obtain in-depth understanding of this infection, the interplay between virulence factors of the pathogen and defense mechanisms of the porcine host needs to be elucidated. However, research has traditionally focused on *either* bacteriology *or* immunology; an unbiased picture of the transcriptional responses can be obtained by investigating both organisms in the same biological sample.

**Results:**

Host and pathogen responses in pigs experimentally infected with *A. pleuropneumoniae* were analyzed by high-throughput RT-qPCR. This approach allowed concurrent analysis of selected genes encoding proteins known or hypothesized to be important in the acute phase of this infection. The expression of 17 bacterial and 31 porcine genes was quantified in lung samples obtained within the first 48 hours of infection. This provided novel insight into the early time course of bacterial genes involved in synthesis of pathogen-associated molecular patterns (lipopolysaccharide, peptidoglycan, lipoprotein) and genes involved in pattern recognition (*TLR4, CD14, MD2, LBP, MYD88*) in response to *A. pleuropneumoniae*. Significant up-regulation of proinflammatory cytokines such as IL1B, IL6, and IL8 was observed, correlating with protein levels, infection status and histopathological findings. Host genes encoding proteins involved in iron metabolism, as well as bacterial genes encoding exotoxins, proteins involved in adhesion, and iron acquisition were found to be differentially expressed according to disease progression. By applying laser capture microdissection, porcine expression of selected genes could be confirmed in the immediate surroundings of the invading pathogen.

**Conclusions:**

Microbial pathogenesis is the product of interactions between host and pathogen. Our results demonstrate the applicability of high-throughput RT-qPCR for the elucidation of dual-organism gene expression analysis during infection. We showed differential expression of 12 bacterial and 24 porcine genes during infection and significant correlation of porcine and bacterial gene expression. This is the first study investigating the concurrent transcriptional response of both bacteria and host at the site of infection during porcine respiratory infection.

**Electronic supplementary material:**

The online version of this article (doi:10.1186/s12864-015-1557-6) contains supplementary material, which is available to authorized users.

## Background

Insight into host-pathogen interaction dynamics is not only essential for the understanding of infection pathogenesis, but also for development of therapeutic interventions for controlling infectious diseases. Focusing on the site of infection when studying the interplay between host and pathogen allows the identification of factors involved in the intricate host-pathogen interactions during early stages of the infection. An unbiased picture of the interdependent transcriptional responses would be obtained by investigating both organisms in the same biological sample. To date, only a few studies employing a simultaneous characterization of concurrent host and pathogen gene expression during mammalian infection have been published [1-4]. Traditionally, research has focused on *either* bacteriology *or* immunology. Technical issues like species adapted methods and the scarcity of pathogen RNA compared to host RNA, all contribute to this conventionally one-sided focus in gene expression studies of infection processes [5]. The emergence of high-throughput qPCR systems, e.g. the BioMark from Fluidigm, offers a platform that is ideal for the focused, hypothesis-driven study of gene expression of even small quantities of RNA [6,7]. The high capacity of such platforms enables the researcher to cover diverse areas – as well as organisms – of interest in the same experimental setup.

Porcine pleuropneumonia, caused by the Gram-negative bacterium *Actinobacillus pleuropneumoniae*, is a contagious respiratory disease often leading to a very rapidly evolving pleuropneumonia. This infection is associated with significantly impaired animal welfare, high morbidity and mortality, resulting in economic losses in the pig production [8,9]. The pig has recently been shown to be a promising animal model for human pneumonia [10-12]. The study of this porcine infection might therefore also provide valuable information with human relevance.

The pathogenesis of pleuropneumonia in pigs is still not fully understood. However, innate pattern recognition receptors (PRRs) [13], inflammatory cytokines [14,15], and proteins involved in depletion of iron available to the bacteria [16] are recognized as important host factors associated with outcome of the infection. The rapidly evolving pleuropneumonia may in severe cases lead to death within 24–36 hours, likely due to the combined effect of tissue damage caused by the bacteria, and a strong proinflammatory immune response [15,17].

Studies have shown that *A. pleuropneumoniae* infection leads to a rapidly and widely disseminated immune response. In the porcine lung, this was demonstrated by the significant regulation of immune related genes in visibly unaffected tissue, as well as in necrotic areas [15]. Also hepatic, splenic, tonsillar, and tracheobronchial lymphatic gene expression was regulated in response to infection, and serum acute phase protein (APP) levels were significantly altered [14,18-20].

The colonization and ability to adapt to local conditions in the host of *A. pleuropneumoniae* is mediated by multiple known and putative infection-associated factors [8,21-24]. Many of the presently known virulence factors of this pathogen have been identified by *in vivo* methods such as signature tagged mutagenesis, *in vivo* expression technology and microarray analysis of bacteria from naturally or experimentally infected pigs [23,25-28].

Among bacterial factors presently considered to be of importance for *A. pleuropneumoniae* virulence are adhesins, iron-acquisition proteins, capsular polysaccharides, and lipopolysaccharides as well as RTX toxins, which are major virulence factors in the genus of *Pasteurellaceae* [8,21,29]. *In vivo* and *in vitro* studies have demonstrated genes involved in cell envelope biogenesis and maintenance to be highly affected during infection [28,30].

So far, studies of gene expression during porcine pleuropneumonia have addressed the pathogen or host separately [13-15,17,23,28,31-33]. Here, we analyzed the relationship between gene expression of *A. pleuropneumoniae* and the porcine host simultaneously in the same lung tissue samples. Porcine and bacterial RNA was extracted, reverse transcribed, and pre-amplified simultaneously. Temporal changes of protein and mRNA coding for host immune factors and mRNA coding for pathogen virulence factors were analyzed during the acute phase of the disease. We used the highly sensitive method of quantitative reverse transcription real-time PCR (RT-qPCR) on a high-throughput chip-based platform allowing the simultaneous analysis of 48 genes and 48 samples – 2304 parallel reactions. Bacterial and porcine mRNA was analyzed on the same chip, and a subset of bacterial and host genes were found to correlate and to be regulated in accordance with infection status. This is the first study revealing the concurrent transcriptional response of bacteria and host at the site of infection during porcine pleuropneumonia.

## Methods

### Animals and infection studies

The experimental infection study is described in detail in reference [23]. All animal procedures were approved by the Danish Animal Experiments Inspectorate under the Ministry of Justice (permit number: 2006/561-1106) and animal experiments were conducted in strict accordance with their guidelines. Briefly, inoculations were carried out using *A. pleuropneumoniae* serotypes 2 (4226) and 6 (7712640) grown overnight on PPLO agar plates (Difco) at 37°C and resuspended in 0.9 % NaCl. Resuspended bacteria were mixed 1:1 with brain heart infusion broth (Difco) supplied with 5 % NAD and used for inoculations. 8-10-weeks-old Danish specific pathogen free piglets were inoculated intranasally with 2 ml bacterial suspension containing 1-2 × 10^8^ CFU. No serotype-specific effects on histopathological findings or on either porcine or bacterial gene expression were observed in subsequent analyses (Additional file 1: Table S1). Therefore, animals were combined to obtain larger sample sizes in each group and to improve statistical power. A direct effect of the broth into the lungs of control animals was not investigated, however in cases of intranasal inoculation with a volume of 2 ml most of the suspension is dispersed in and absorbed from the upper respiratory system and does not reach the lungs as a bolus. Furthermore, control animals were housed and fed similarly to infected animals and handled by the same animal caretakers. Samples from uninfected control animals (n = 6 for protein, n = 7 for RNA), and animals sacrificed 6 hours (h) post inoculation (p.i.) (n = 6), 12 h p.i. (n = 5), 24 h p.i. (n = 8), and 48 h p.i. (n = 5) were included. Lung tissue samples of approx. 0.5 cm × 0.5 cm × 0.5 cm cubes from pulmonary lesions were manually dissected and preserved in RNAlater (Ambion) and stored at −20°C. Samples for laser capture microdissection (LCM) were snap frozen in liquid nitrogen (approx. 1 cm × 1 cm × 0.5 cm) and stored at −80°C. For histology, lung tissues were fixed in 10 % neutral buffered formalin and slides were processed by routine methods and stained with haematoxylin and eosin (HE).

### Laser capture microdissection (LCM)

Pleuropneumonic lung tissue from animals sacrificed 6 h and 24 h p.i. was sectioned (8 μm) using a Leica CM1850 cryostat (Leica Microsystems) at −24°C and mounted on 0.17 mm PEN MembraneSlides (Carl Zeiss MicroImaging). Preparation of MembraneSlides comprised rinsing once in 0.5 % NaOH and twice in RNase free water, drying at 46°C for 30 minutes, and treating in a UV Crosslinker (AH diagnostics) for 30 minutes. Once mounted on the MembraneSlides, tissue sections were stored at −80°C. Bacteria were stained using immunofluorescence. Rabbit anti-*A. pleuropneumoniae* serotype 5b strain L20 IgG (produced in-house, National Veterinary Institute) was biotinylated using ChromaLink Biotin Labeling Kit (Solulink). Biotinylated antibodies were used in combination with streptavidin-conjugated fluorescent dye (Cy3) in Arcturus HistoGene LCM Immunofluorescence Staining Kit (Life Technologies). Biotinylated antibodies were applied in a concentration of 100 μg/ml, and streptavidin-conjugated Cy3 was applied in a 1:100 dilution.

Control stainings of *A. pleuropneumoniae* serotypes 2 (4226) and 6 (7712640) were performed to ensure that IgG raised against serotype 5b strain L20 also recognized these serotypes. Immunofluorescence staining was followed immediately by LCM, carried out using a PALM MicroBeam system (P.A.L.M. Microlaser Technologies AG), comprised of an Axiovert 200 M microscope (Carl Zeiss) with a 100-W Hg lamp, a 40×/1.30 oil Fluar objective (Carl Zeiss), filter set XF53 (Omega Optical), and PALM RoboSoftware v. 4 SP2 (P.A.L.M. Microlaser Technologies AG). Fluorescently labeled bacterial colonies and surrounding tissue were microdissected and catapulted into the lid of a 0.2 ml tube containing 25 μl extraction buffer from the Arcturus PicoPure RNA Isolation Kit (Life Technologies). LCM was performed in triplicate for each animal.

### RNA isolation

Experimental practice and reporting has been performed according to the minimum information for publication of quantitative real-time PCR experiments (MIQE) guidelines [34]. Manually collected samples of approx. 300 mg were homogenized using a gentleMACS Dissociator (Miltenyi Biotec) in M tubes (Miltenyi Biotec) containing Qiazol (Qiagen). RNA was extracted using RNeasy Lipid Tissue Midi Kit (Qiagen), according to manufacturer’s specifications. RNA integrity numbers (RIN) were determined using an Agilent 2100 Bioanalyzer, Agilent RNA 6000 Nano Chips, and Agilent RNA 6000 Nano reagents (Agilent Technologies, USA). RIN ranged from 4.7 to 8.2, mean RIN was 6.5, which was accepted for RT-qPCR in accordance with previous review of the effect of RNA integrity on RT-qPCR performance [35]. RNA purity and concentration were determined using a NanoDrop ND-1000 UV spectrophotometer (Thermo Scientific). RNA concentrations ranged from 107 to 1612 ng/μl, mean RNA concentration was 554 ng/μl. A260/280 and A260/230 ratios were above 2 for all samples. RNA extracts were stored at −80°C.

Extraction of RNA from microdissected tissue was performed immediately after LCM, using Arcturus PicoPure RNA Isolation Kit (Life Technologies) according to the manufacturer’s specifications. RNA was eluted in 20 μl elution buffer (supplied in kit). RNA concentrations were determined using a NanoDrop ND-1000 UV spectrophotometer, and ranged from ~1 to ~14 ng/μl. RIN of LCM samples was not measured due to the small amount of total RNA; all material was saved for use in the reverse transcription. RNA extracts were stored at −80°C.

### Reverse transcription, pre-amplification and exonuclease treatment

QuantiTect Reverse Transcription Kit (Qiagen) was used for cDNA synthesis, employing a mix of oligo-dT and random primers. Two technical replicates of cDNA synthesis were made from each RNA sample, both manually dissected and LCM. Reverse transcription of RNA from manually dissected material was performed using 500 ng of RNA for each reaction. The amount of RNA applied in reverse transcription of LCM samples varied due to very low and varying RNA concentrations obtained in the LCM RNA extraction procedure. Non-reverse transcriptase controls were likewise prepared for both manually dissected and LCM samples, and included in the following pre-amplification and qPCR. cDNA samples were pre-amplified using 5 μl TaqMan PreAmp Master Mix (Life Technologies), 2.5 μl 200 mM mix of the porcine and bacterial qPCR primers and 2.5 μl diluted cDNA (1:8 in low EDTA TE-buffer). Pre-amplification was initiated by 10 min hot start at 95°C, followed by 14 (manually dissected) or 17 cycles (LCM) of denaturing for 15 s at 95°C and annealing/extending for 4 min at 60°C. Pre-amplified cDNA was treated with Exonuclease I (*E. coli*) (New England Biolabs) for 30 min at 37°C followed by 15 min at 80°C.

### Assay design

Primers were designed in the Primer3 software (http://bioinfo.ut.ee/primer3-0.4.0/) for both bacteria (17) and host (31) genes using similar criteria for T_m_ and amplicon length as described in reference [14]. Sequences of porcine and bacterial primers as well as PCR efficiencies (based on three separate 5-fold dilution series of pooled cDNA samples, run on a separate qPCR chip), T_m_, and amplicon lengths can be found in Additional file 2: Table S2.

All investigated genes are summarized in Table 1. Bacterial assays fall into two overall categories: 1) genes involved in cell wall membrane biogenesis, potentially interacting with the innate immune response of the host: *kdsB* (3-deoxy-manno-octulosonate cytidylyltransferase), *wzxE* (Lipopolysaccharide biosynthesis protein), *ompA* (Outer membrane protein P5), *mltC* (Murein transglycosylase C), *palA* (Outer membrane protein), *tolA* (cell envelope integrity inner membrane protein), *wecC* (UDP-N-acetyl-D-mannosamine dehydrogenase) and *ompP4* (Lipoprotein E). 2) known or potential virulence factors expected to be expressed *in vivo* during infection: two genes, *afuB* (ferric transport system permease protein) and *hgbA* (hemoglobin-binding protein), encoding proteins involved in iron uptake, a function considered to be important for bacterial survival in the host [8,36]. *apxIIA* (RTX-II toxin determinant A) was selected due to the fact that exotoxin production is a major virulence factor of *A. pleuropneumoniae* [37]; only one toxin gene was included due to the limited amount of space on the chip. Two potential virulence factors involved in adherence and competence were included: *comEA* (fibronectin adhesion protein) and *csgG* (putative lipoprotein) [38,39]. *csgG* has previously been observed to be up-regulated *in vivo* in pig lung during the acute phase of disease and during biofilm formation [28,40], and its homologue proved to be immunogenic in pathogenic *Haemophilus parasuis* [41]. *mgsA* was included as methylglyoxal appears to play a key role in the physiology of intracellular pathogens [42] and *mgsA* seems to be important for *Haemophilus influenzae* survival in a murine host [43]. The chaperone gene *dnaK*, encoding HSP70, was included as it has previously been reported as immunoreactive in convalescent sera from pigs naturally infected with *A. pleuropneumoniae* [44]. Table 1 states the functional grouping of all investigated porcine genes and thereby also the rationale behind including these genes in the study. Porcine assays were focused around uncovering the pathogen recognition in the host lung, the inflammatory response and immune modulation to control the infection, and nutrient sequestration (iron) from the invading pathogen.

**Table 1:** **Relative expression levels of porcine and bacterial genes**

***Gene***	**Gene product**	**Control** ^**3**^	**6 h p.i.** ^**3**^	**12 h p.i.** ^**3**^	**24 h p.i.** ^**3**^	**48 h p.i.** ^**3**^	***P*** **value** ^**4**^
**Porcine** ^**1**^ **– Genes involved in pattern recognition**							
*TLR4*	Toll-like receptor 4	1.0 ± 0.1	12.6 ± 1.6	8.3 ± 2.6	4.1 ± 1.2	3.7 ± 0.6	9.00E-08
*CD14*	Cluster of differentiation 14	1.0 ± 0.2	8.7 ± 0.8	7.6 ± 2.1	4.5 ± 1.1	4.1 ± 0.4	8.00E-08
*MD2*	Myeloid differentiation protein-2	1.0 ± 0.1	3.4 ± 0.4	3.2 ± 0.4	2.0 ± 0.3	2.0 ± 0.4	1.06E-05
*LBP*	Lipopolysaccharide-binding protein	1.0 ± 0.4	8.8 ± 1.9	8.6 ± 4.4	8.6 ± 2.0	24.7 ± 4.5	3.38E-05
*MYD88*	Myeloid differentiation primary response 88	1.0 ± 0.1	5.2 ± 0.7	4.6 ± 1.5	1.8 ± 0.3	2.5 ± 0.6	7.25E-06
*IRF3*	Interferon regulatory factor 3	1.0 ± 0.1	1.4 ± 0.2	1.7 ± 0.4	1.0 ± 0.1	1.0 ± 0.1	0.021
*SFTPA*	Surfactant protein A	1.0 ± 0.1	0.6 ± 0.1	0.7 ± 0.2	0.7 ± 0.2	0.3 ± 0.2	0.014
*SFTPD*	Surfactant protein D	1.0 ± 0.1	0.6 ± 0.1	1.0 ± 0.4	0.6 ± 0.1	0.5 ± 0.3	NS
**Porcine** ^**1**^ **– Genes involved in involved in iron metabolism**							
*CD163*	CD163 molecule	1.0 ± 0.1	0.8 ± 0.1	3.0 ± 1.1	2.1 ± 0.2	3.0 ± 0.7	0.022
*HP*	Haptoglobin	1.0 ± 0.2	2.2 ± 0.1	2.1 ± 0.2	1.2 ± 0.2	1.2 ± 0.5	0.004
*TF*	Transferrin	1.0 ± 0.1	0.4 ± 0.1	0.5 ± 0.2	0.3 ± 0.1	0.1 ± 0.0	1.31E-04
*LTF*	Lactotransferrin	1.0 ± 0.6	10.2 ± 5.9	30.4 ± 22.0	10.5 ± 6.0	7.1 ± 3.5	NS
**Porcine** ^**1**^ **– Cytokines involved in innate immune modulation and inflammation**							
*IL1B*	Interleukin-1 β	1.0 ± 0.2	414 ± 50	253 ± 93	175 ± 41	209 ± 27	7.20E-07
*IL1RN*	Interleukin-1 receptor antagonist	1.0 ± 0.2	80.1 ± 12.9	53.4 ± 21.2	40.7 ± 9.7	37.8 ± 3.6	7.50E-07
*IL6*	Interleukin-6	1.0 ± 0.1	1024 ± 67	256 ± 99	80.2 ± 29.1	48.9 ± 22.3	6.00E-08
*IL8*	Interleukin-8	1.0 ± 0.2	261 ± 19	181 ± 69	91.0 ± 26.1	104 ± 19	1.28E-06
*IL17A*	Interleukin-17A	1.0 ± 0.3	325 ± 54	116 ± 55	46.1 ± 13.4	21.5 ± 4.1	1.49E-05
*IL18*	Interleukin-18	1.0 ± 0.1	3.7 ± 0.4	7.1 ± 2.8	6.5 ± 2.1	6.0 ± 0.8	1.51E-04
*IFNG*	Interferon-gamma	1.0 ± 0.2	3.1 ± 0.4	1.1 ± 0.3	1.0 ± 0.2	0.3 ± 0.1	1.19E-05
*TNF*	Tumor necrosis factor	1.0 ± 0.1	4.8 ± 0.6	2.5 ± 0.5	2.0 ± 0.7	1.8 ± 0.3	3.20E-04
*CSF2*	GM-CSF	1.0 ± 0.1	4.4 ± 0.6	2.6 ± 0.9	1.4 ± 0.3	0.6 ± 0.3	4.69E-05
**Porcine** ^**1**^ **– Genes involved in the complement system and other functions**							
*C3*	Complement component 3	1.0 ± 0.1	1.4 ± 0.1	1.0 ± 0.1	0.9 ± 0.1	0.7 ± 0.2	0.015
*CFB*	Complement factor B	1.0 ± 0.1	5.6 ± 0.4	3.4 ± 0.9	2.7 ± 0.4	2.9 ± 1.1	1.28E-05
*CFD*	Complement factor D	1.0 ± 0.1	1.4 ± 0.2	1.1 ± 0.1	1.1 ± 0.1	0.8 ± 0.1	NS
*MASP2*	Mannan-binding lectin serine peptidase 2	1.0 ± 0.1	0.4 ± 0.0	0.5 ± 0.2	0.3 ± 0.1	0.2 ± 0.0	1.14E-04
*SAA*	Serum amyloid A	1.0 ± 0.2	15.3 ± 2.3	20.4 ± 11.0	68.9 ± 19.6	110.2 ± 19.6	7.57E-06
*TNFAIP3*	Tumor necrosis factor, alpha-induced protein 3	1.0 ± 0.1	17.5 ± 1.7	7.7 ± 2.6	4.1 ± 1.0	3.7 ± 0.4	8.00E-07
*GZMB*	Granzyme B	1.0 ± 0.5	3.7 ± 1.6	1.0 ± 0.5	1.3 ± 0.3	0.4 ± 0.1	0.0069
**Bacterial** ^**2**^ **– Genes involved in cell wall membrane biogenesis**							
*kdsB*	3-deoxy-manno-octulosonate cytidylyltransferase	NA	2.6 ± 0.5	1.7 ± 0.2	1.2 ± 0.1	1.0 ± 0.1	7.97E-04
*wzxE*	Lipopolysaccharide biosynthesis protein	NA	1.7 ± 0.1	1.4 ± 0.1	1.2 ± 0.1	1.0 ± 0.1	1.50E-03
*ompA*	Outer membrane protein P5	NA	1.0 ± 0.1	2.5 ± 0.3	3.6 ± 0.5	3.6 ± 0.7	3.11E-05
*mltC*	Murein transglycosylase C	NA	1.9 ± 0.2	1.7 ± 0.1	1.3 ± 0.1	1.0 ± 0.1	8.20E-05
*palA*	Outer membrane protein	NA	1.7 ± 0.1	1.4 ± 0.2	1.0 ± 0.1	1.1 ± 0.1	9.01E-04
*tolA*	Cell envelope integrity inner membrane protein	NA	1.0 ± 0.1	1.5 ± 0.1	1.5 ± 0.2	1.6 ± 0.1	9.99E-03
*wecC*	UDP-N-acetyl-D-mannosamine dehydrogenase	NA	2.2 ± 0.1	1.8 ± 0.2	1.5 ± 0.1	1.0 ± 0.2	2.72E-04
*ompP4* ^*5*^	Lipoprotein E	NA	4.2 ± 0.6	2.8 ± 0.7	1.4 ± 0.3	1.0 ± 0.2	4.07E-04
**Bacterial** ^**2**^ **– Genes involved in iron uptake**							
*afuB*	Ferric transport system permease protein fbpB	NA	3.9 ± 0.4	2.6 ± 0.3	1.6 ± 0.2	1.0 ± 0.1	2.33E-06
*hgbA*	Hemoglobin-binding protein	NA	1.3 ± 0.1	1.0 ± 0.1	1.0 ± 0.2	1.0 ± 0.2	NS
**Bacterial** ^**2**^ **– Gene involved in exotoxin production**							
*apxIIA*	RTX-II toxin determinant A	NA	7.0 ± 0.7	3.0 ± 0.7	2.3 ± 0.4	1.0 ± 0.3	5.16E-06
**Bacterial** ^**2**^ **– Genes involved in adhesion and competence**							
*comEA*	Fibronectin adhesion protein	NA	5.8 ± 0.8	2.8 ± 0.5	1.5 ± 0.4	1.0 ± 0.2	5.33E-05
*csgG*	Putative lipoprotein	NA	1.2 ± 0.1	1.2 ± 0.2	1.0 ± 0.2	1.0 ± 0.2	NS
**Bacterial** ^**2**^ **– Genes other functions**							
*dnaK*	Chaperone protein Dna	NA	2.9 ± 0.4	2.6 ± 1.3	2.0 ± 0.8	1.0 ± 0.5	NS
*mgsA*	Methylglyoxal synthase	NA	1.8 ± 0.2	1.3 ± 0.2	1.0 ± 0.1	1.4 ± 0.2	0.027

^1^Sample sizes: Control: n = 7; 6 h p.i.: n = 6; 12 h p.i.: n = 5; 24 h p.i.: n = 8; 48 h p.i.: n = 5.

^2^Number of data points: 6 h p.i.: n = 6; 12 h p.i.: n = 4; 24 h p.i.: n = 7; 48 h p.i.: n = 5.

^3^Expression is given ± SEM.

^4^P values are included for genes that were differentially expressed during the course of the study (ANOVA, P < 0.05). NS = not significant.

^5^Gene also involved in iron uptake (heme acquisition).

### High-throughput qPCR

High-throughput qPCR was applied using Dynamic Array IFC 48.48 chips (Fluidigm) for the BioMark HD System (Fluidigm), TaqMan Gene Expression Master Mix (Life Technologies), EvaGreen 20X (VWR Bie & Berntsen), and gene specific primers as previously described [12]. qPCR was initiated by 2 min at 50°C and 10 min at 95°C, followed by 35 cycles of denaturing for 15 s at 95°C and annealing/elongation for 1 min at 60°C. Melting curves were generated after each run to confirm the presence of a single PCR product (from 60°C to 95°C, increasing 1°C/3 s). Non-template controls and three interplate calibrators were included on each chip.

### Protein extraction and ELISA

Lung tissue was extracted by homogenization with 1 ml 0.1 M Tris/HCl pH 7.2, 0.1 M NaCl, plus protease inhibitor cocktail Complete Ultra from Roche (1 tablet per 10 ml buffer) per 100 mg tissue, using 0.2 to 0.5 g of tissue. Homogenization was performed at room temperature with a gentleMACS Dissociator from Miltenyi Biotec using M-tubes (Miltenyi Biotec) and the RNA_02.01 M Tube protocol in which the tissue is blended for 81 seconds. This was followed by incubation on rocking table overnight at 4°C. Finally, samples were centrifuged for 20 minutes at 5,500 x G and the supernatant was retrieved and used for analysis of cytokines by ELISA.

IL-6, IL-1β, and IL-8 concentrations were determined by sandwich ELISAs from R&D Systems (Duoset DY686, Duoset DY681 and Duoset DY535, respectively) using a calibrated porcine IL-6/IL-1β/IL-8 standard, respectively, supplied by the manufacturer. Porcine IFN-γ was quantified by sandwich ELISA as described in reference [45], using a mouse monoclonal anti-pig IFN-γ antibody (clone P2F6, Pierce Biotechnology) for catching and a biotinylated mouse monoclonal anti-pig IFN-γ antibody (clone P2C11, BD Biosciences Pharmingen) and peroxidase-conjugated streptavidin (Invitrogen) for detection. Samples were run in duplicates in a dilution of 1:2.

Lung tissue extract cytokine concentrations were standardized in the following way: the absorbance at 280 nm was determined for each sample and used to normalize each sample value individually by multiplying with the correction factor: [A280 (mean, all samples)]/[A280 (sample)]. The detection limit for all ELISAs was determined as the lowest concentration in the standard row multiplied by the dilution factor employed for the samples.

### Data processing and statistics

qPCR data was inspected using Fluidigm Real-Time PCR Analysis software (v. 3.0.2), and pre-processed and analyzed using GenEx software (v. 5.3.7) (MultiD). Data was corrected for PCR efficiencies for each primer assay. As three Dynamic Array IFC 48.48 chips were necessary to accommodate all samples, data was normalized with three interplate calibrator samples included on all chips. The resulting data was then normalized using reference gene expression. A panel of eight porcine reference genes had previously been tested on the same *A. pleuropneumoniae* infected lung material as applied in this study (data not shown), and using the algorithms geNorm [46] and NormFinder [47], the genes beta-actin (*ACTB)* and peptidylprolyl isomerase A (*PPIA*) were found to be the most stably expressed. Porcine qPCR data was normalized with *PPIA. csrA* (carbon storage regulator) and *manB* (phosphomannomutase) were used for normalization of bacterial qPCR data, as these had previously been validated as suitable bacterial reference genes for the tissue applied in the present study [23]. cDNA replicates were averaged after reference gene normalization. For each qPCR assay (gene) all C_q_ values were converted to relative quantities by calculating 2^(highest_assay_Cq – actual_sample_Cq)^, thus giving the sample with the highest C_q_ (lowest gene expression) a value of 1 and all other samples values >1. The mean values for each group was computed, and in order to easily visualize gene expression fold changes between time points, scaling of data was performed. Porcine gene expression at all time points p.i. was displayed relative to expression in controls (mean expression in control animals set to 1). For each bacterial gene, the time point with the lowest expression was set to 1, and all other time points displayed relative to this. Data were log_2_ transformed to approach a normal distribution prior to ANOVA or *t*-test, and genes were considered to be differentially expressed if *P* < 0.05, and if the expression changed ≥2-fold over the course of infection. Pearson’s correlation coefficient was used to determine the temporal correlation of 1) expression of porcine and bacterial genes, 2) of porcine gene expression measured in manually dissected samples and LCM samples, and 3) of mRNA and protein levels of cytokines. Correlations were considered significant at level of *P* < 0.05 (TDIST function in Excel). Log_2_ transformed bacterial expression data were further analyzed by principal component analysis (PCA), included in the GenEx software, in order to describe the overall structure of the dataset and to identify homogeneous subgroups of samples.

## Results

### Histopathology

**Figure 1:**
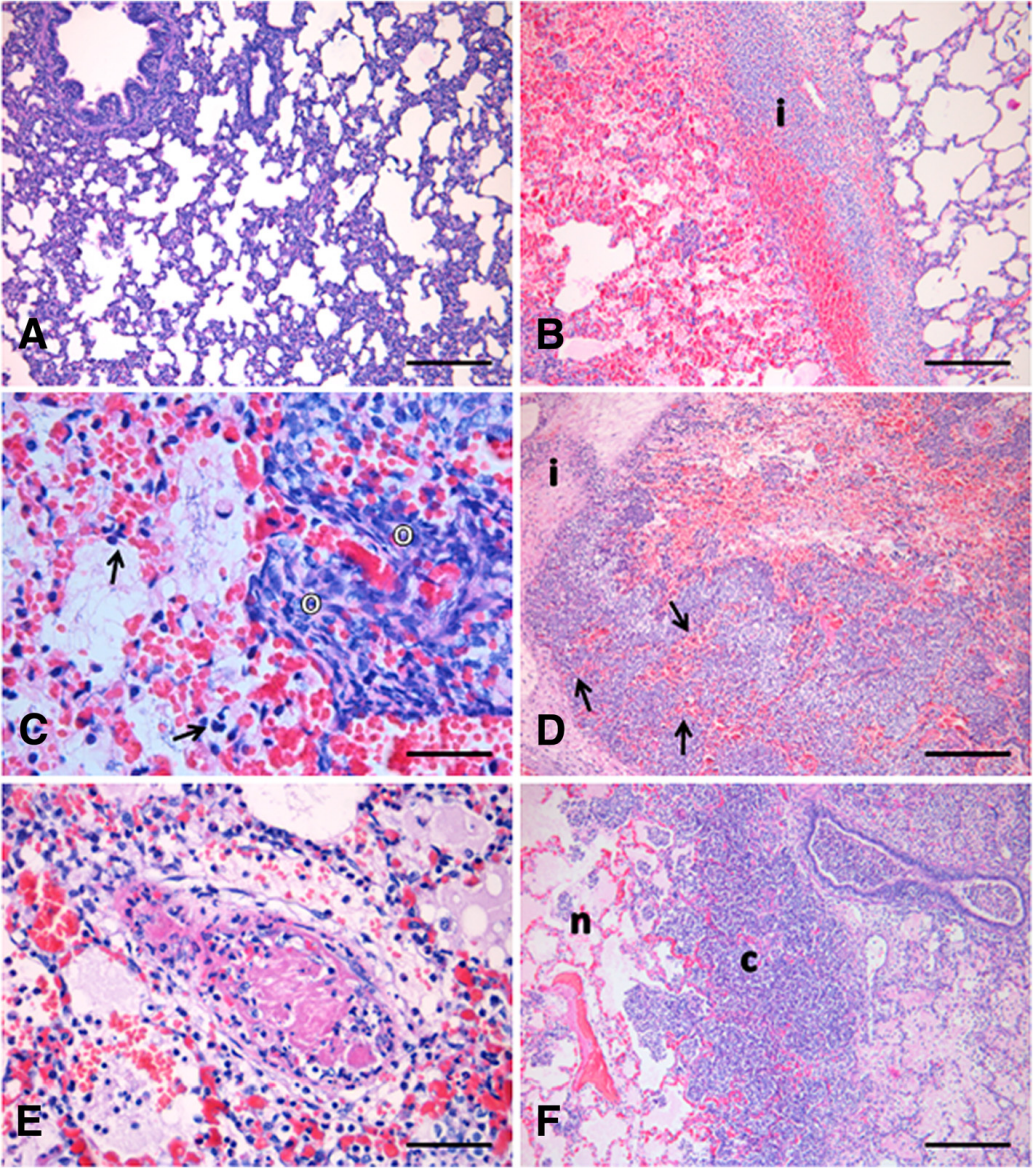
HE-stained lung sections. **(A)** Lung section from a control pig with mild thickening of the alveolar septa. Scale bar 200 μm; **(B-F)** Lung sections from pigs inoculated with *A. pleuropneumoniae*. **(B)** 6 h p.i.: An interlobular septa [i] with bleeding, oedema and infiltration of neutrophils, separates a non-affected lobule and a lobule with hyperemia, bleeding, oedema, infiltration of neutrophils, bacteria and fibrin. Scale bar 200 μm; **(C)** 6 h p.i.: Necrosis and degeneration of alveolar septal cells (arrows), and presence of oat-shaped cells [o]. Scale bar 30 μm; **(D)** 12 h p.i.: Increased cellular infiltration along an interlobular septa [i] and coagulative necrosis of the alveolar septa (arrows). Scale bar 200 μm; **(E)** 24 h p.i.: Fibrinoid necrosis of a blood vessel. Scale bar 40 μm; **(F)** 48 h p.i.: Lobule affected by coagulative necrosis [n] enclosed by dense fringes of neutrophils and mononuclear cells [c]. Scale bar 200 μm.

At 6 h p.i. the lung tissue was affected by acute inflammation, characterized by hyperemia, bleeding, oedema, influx of neutrophils, numerous bacteria, fine threads of fibrin, and degeneration or necrosis of the alveolar septal cells (Figure 1B and C). Among the infiltrating neutrophils there were necrotic and activated neutrophils (i.e. “oat-shaped cells”). Lesions at 12, 24 and 48 h p.i. resembled those described at 6 h p.i. but with some variations. At 12 h p.i. the cellular infiltration had expanded and still consisted mainly of neutrophils, but with increasing numbers of mononuclear cells (Figure 1D). The fibrin had aggregated into cloths and necrotizing vasculitis was observed. At 24 h p.i. the number of inflammatory cells had increased, most lobules were severely affected by coagulative necrosis, and fibrinoid necrotizing vasculitis was seen (Figure 1E). After 48 h p.i. the lesions had developed further and the large areas of coagulative necrosis were enclosed by dense fringes of cells including many oat-shaped cells (Figure 1 F). Histopathological examination of lung tissue from the control animals revealed mild thickening of the alveolar septa (Figure 1A). The observation of thickened alveolar septa in the control pigs is a common finding in conventional pigs and is considered to be a pulmonary response to environmental factors associated with swine production [48]. Two animals differed from the general descriptions outlined above (one pig at 12 h p.i. and one pig at 24 h p.i.), by having mainly moderate non-suppurative interstitial pneumonia and only moderate focal acute lesions. In addition, in these two specific animals it was not possible to detect bacterial gene expression using RT-qPCR, and IL-1β and IL-8 protein concentrations were below the limit of detection. Collectively, these observations suggest that bacterial infection was comparatively less efficiently established in these two animals.

### Concurrent gene expression patterns in lung tissue: Host pattern recognition receptor genes and pathogen-associated molecular pattern genes

**Figure 2:**
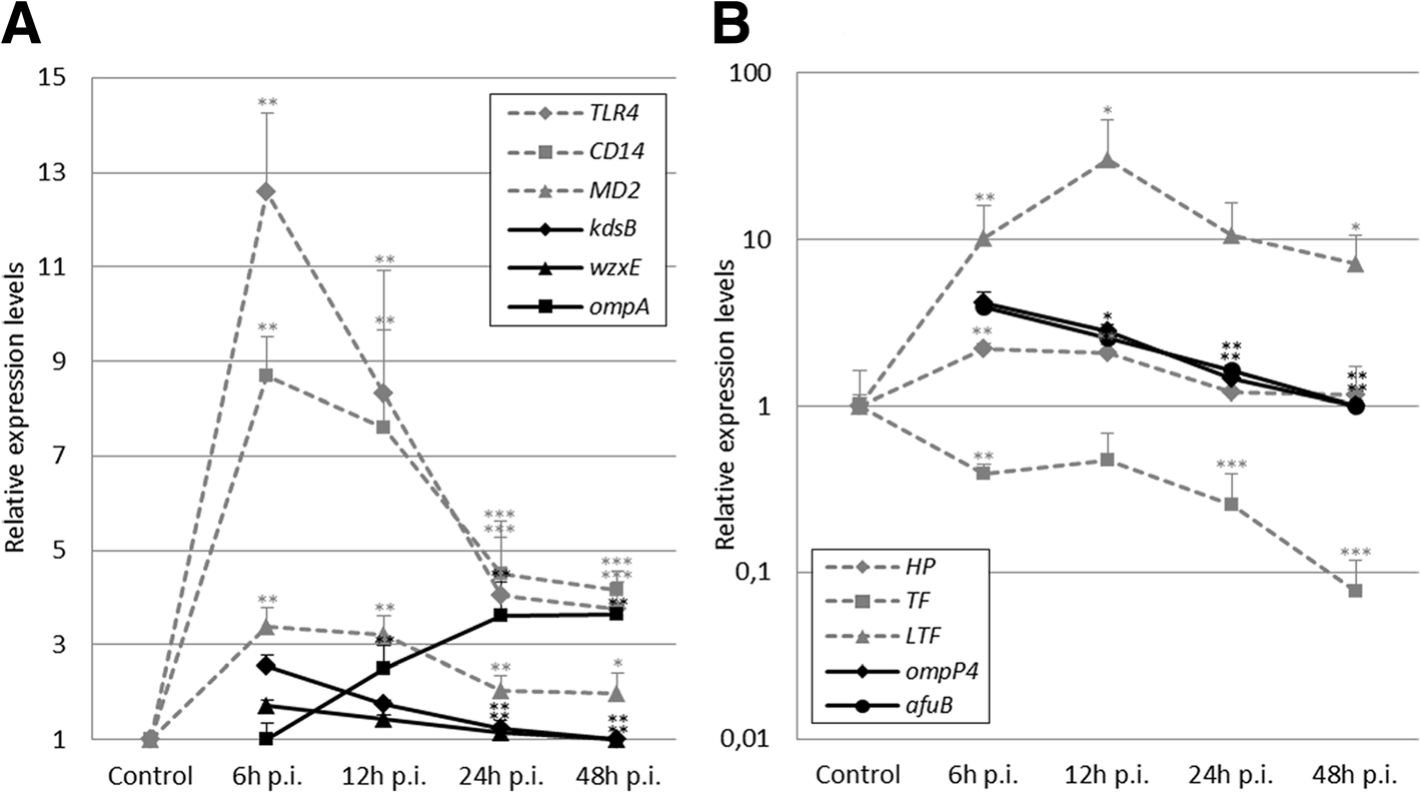
Temporal development of porcine and bacterial gene expression profiles. Porcine data (dotted gray) is shown as mean expression relative to control animals; bacterial data (black) is shown as mean expression relative to the time group with the lowest expression. Sample sizes are given in Table 1. SEM is depicted by error bars. *P < 0.05, **P < 0.01, ***P < 0.001 (t-test or Mann–Whitney if normal distribution could not be demonstrated) relative to control (porcine) and lowest expression (bacterial). **A**) expression of porcine genes involved in recognition of LPS, and bacterial genes involved in LPS synthesis (*kdsB* and *wzxE*) and *ompA*. **B**) expression of porcine genes involved iron binding and transportation and bacterial genes involved iron acquisition. Note y-axis in log-scale.

Expression of the porcine genes toll-like receptor 4 (*TLR4*), cluster of differentiation 14 (*CD14*), and myeloid differentiation protein-2 (*MD2*), all involved in recognition of Gram-negative lipopolysaccharide (LPS), increased and peaked at 6 h p.i. (Figure 2a). Likewise, LPS-binding protein (*LBP*) and myeloid differentiation primary response 88 (*MYD88*), also involved in TLR4 signaling, were significantly up-regulated after infection (Table 1). Interferon regulatory factor (*IRF3*) was significantly up-regulated in a temporal pattern highly similar to the other TLR4 signaling related genes, but never more than 1.7-fold (Table 1). In the pathogen, several genes involved in synthesis of the outer bacterial membrane components were differentially expressed during infection (Figure 2a; Table 1). A significant correlation was seen between expression patterns of bacterial genes involved in LPS synthesis (*kdsB* and *wzxE*) and host genes involved in the recognition of and response to LPS (*TLR4, CD14, MD2,* and *MYD88*, Table 2). Moreover, genes related to TLR4-mediated signal transduction were also significantly correlated to several bacterial genes involved in peptidoglycan synthesis (*palA, wecC*, and *mltC*, Table 2). *TLR4, CD14, MD2, kdsB*, and *wzxE* (Figure 2a, Table 1), as well as *mltC, wecC*, and *palA* (Table 1) were all highly expressed at 6 h p.i. After that, their expression decreased and was found to be lowest at 24 h and 48 h p.i. In contrast, *ompA* (Figure 2a) and *tolA* (Table 1) gradually increased from 6 h to 48 h p.i. and thus correlated negatively with host gene expression of *TLR4* and associated components. *LBP* stands out from the remaining TLR4-related genes as its expression increased slowly during infection, with a maximal response at 48 h p.i. (Table 1).

**Figure 3:**
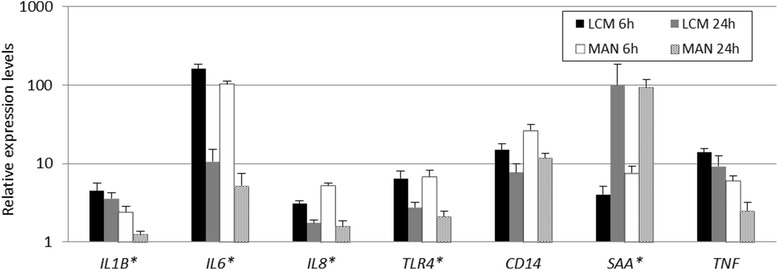
Relative gene expression at 6 h and 24 h p.i. in microdissected samples. RNA was obtained from porcine cells in the infection loci (LCM) and manually dissected lung material (MAN). Note y-axis in log-scale. Error bars depict SEM. * indicate high correlation between LCM and MAN samples (Pearson’s r > 0.72, *P* < 0.05).

### Concurrent gene expression patterns in lung tissue: Host and bacterial genes involved in iron sequestration and acquisition

Expression in infected pig lungs of genes encoding haptoglobin (*HP*), lactotransferrin (*LTF*), and transferrin (*TF*), all involved in binding and transport of iron, were significantly changed at several time points p.i. (Figure 2b, Table 1). *LTF* was highly up-regulated during infection, peaking at 12 h p.i. *HP* was less up-regulated, with a 2-fold increase of expression levels at 6 and 12 h p.i., and *TF* was down-regulated at 6 h p.i. and continued to decrease until 48 h p.i. The gene encoding *CD163*, a receptor for hemoglobin-haptoglobin complexes, was also significantly up-regulated in response to infection (Table 1). Expression of bacterial genes involved in iron acquisition from the host environment was significantly induced during infection (Figure 2b, Table 1). The highest measured expression of *ompP4* and *afuB* occurred at 6 h p.i., followed by a decreased expression of both genes until 48 h p.i. No differential expression of hemoglobin-binding protein (*hgbA)* was detected (Table 1). Significant positive correlations were seen between expression of the porcine genes *HP* and *TF* and bacterial genes *afuB* and *ompP4* from 6 to 48 h p.i. (Table 2).

### Porcine inflammatory response; bacterial adhesion and cytotoxin

As expected, moderate (2–10 fold) to strong (>10 fold) up-regulation of proinflammatory cytokines was seen early in the response to infection (Table 1). The strongest responding cytokine was *IL6*, displaying >1,000-fold increase in expression at 6 h p.i. relative to uninfected controls. Also *IL1B, IL8*, and *IL17A* were highly up-regulated (>100 fold) at 6 h p.i. More moderate regulation was seen for *IL18, TNF*, *IFNG,* granulocyte-macrophage colony stimulation factor (*CSF2*), and the inflammatory marker *TNFAIP3*. These displayed maximum up-regulation less than 10-fold within the first 48 h of infection. The anti-inflammatory cytokine *IL1RN* was also differentially expressed in response to infection, following the same expression pattern as *IL1B* and *IL8*. As seen in Table 2, the expression of cytokines involved in Th17 differentiation (*IL6, IL1B, IL17*), was highly correlated with expression of bacterial genes involved in synthesis of the outer membrane (OM) as well as *apxIIA* and *comEA*. Correlation was for the most part significant and positive except for correlation with *ompA* and *tolA*, which was negative. The only cytokine to deviate from this pattern was *IL18*, which did not correlate with the expression of any analyzed bacterial genes. The expression of serum amyloid A (*SAA*) was also found to respond to infection (Table 1). *SAA* was most highly expressed at 48 h p.i., i.e. later than the peak expression of the proinflammatory cytokines.

*apxIIA*, a pore-forming exotoxin and major virulence determinant for *A. pleuropneumoniae,* and *comEA*, a fibronectin adhesion protein were the two most strongly regulated bacterial genes in the present study (Table 1). The highest observed expression for both genes occurred at 6 h p.i. and then expression of *apxIIA* and *comEA* continued to decrease until 48 h p.i.

### Principal component analysis

PCA of all bacterial gene expression data discriminated between samples belonging to different time groups. Bacterial expression profiles from 6 h p.i. resulted in more well-defined groups with less variation compared to later time points (Additional file 3: Figure S1).

### Laser capture microdissection

Expression of porcine genes involved in LPS recognition and inflammation were also analyzed in microdissected lung samples at time points 6 h and 24 h p.i. (Figure 3). This was performed to compare the host expression profiles obtained from the more heterogeneous manually dissected lung tissue, to the expression from host tissue surrounding the colonies of *A. pleuropneumoniae*. Porcine genes that displayed high expression in the high-throughput gene expression analysis of manually dissected lung material were selected for analysis in LCM samples. Expression of *TLR4, IL1B, IL6, IL8*, and *SAA* in manually dissected material and LCM material was highly correlated (Pearson’s r > 0.72, *P* < 0.05); *CD14* and *TNF* expression was also positively correlated in the two sample types, although not significantly. Even though more variation was seen in the microdissected samples, we could confirm that expression determined in manually dissected lung tissue concurred with expression patterns obtained from porcine cells directly neighboring the infecting *A. pleuropneumoniae* colonies (Figure 3).

### Cytokine detection by ELISA

Cytokines were successfully detected in the same tissue as subjected to gene expression analysis. Lung tissue protein concentrations of IL-1β, IL-6, IL-8, and IFN-γ were determined in uninfected controls as well as infected animals at 6, 12, 24, and 48 h p.i. (Table 3). At 6 h p.i. protein levels of all four inflammatory cytokines were significantly increased compared with the control group. IL-1β and IL-8 were significantly elevated at 6 h p.i. and remained so until 48 h p.i. IL-6 was found to be significantly increased at 6 h and 24 h p.i. and borderline significant (*P* = 0.05) at 48 h p.i. IFN-γ was significantly increased at 6 h p.i. in a small number of samples IL-1β and IL-8 protein concentrations were below the limit of detection. This was especially the case for uninfected control lungs, but also in two infected animals (one from 12 h p.i and one from 24 h p.i.). These coincided with animals in which bacterial gene expression could not be determined either, and both animals only had mild and non-suppurative interstitial pneumonia based on histopathological examinations.

**Table 2:** **Correlation between bacterial and porcine gene expression by Pearson product-moment correlation coefficient**

		**TLR4-related signalling**					**Iron binding**			**Inflammatory cytokines**						
		***TLR4***	***CD14***	***MD2***	***LBP***	***MyD88***	***HP***	***TF***	***CD163***	***IL1B***	***IL6***	***IL8***	***IL17A***	***IL18***	***TNF***	***IFNG***
Capsular and outer membrane	*kdsB*	**0.64**	**0.44**	**0.42**	-0.21	**0.49**				**0.58**	**0.70**	**0.50**	**0.68**	-0.05	**0.56**	**0.71**
	*wecC*	**0.65**	**0.58**	**0.49**	*-0.45*	**0.52**				**0.57**	**0.76**	**0.60**	**0.70**	0.10	**0.65**	**0.69**
	*ompA*	*-0.62*	*-0.51*	*-0.48*	0.31	*-0.54*				*-0.55*	*-0.76*	*-0.62*	*-0.65*	0.05	*-0.66*	*-0.66*
	*wzxE*	**0.80**	**0.69**	**0.60**	-0.17	**0.61**				**0.74**	**0.78**	**0.63**	**0.75**	0.11	**0.65**	**0.65**
	*mltC*	**0.62**	**0.51**	**0.47**	-0.28	**0.45**				**0.52**	**0.73**	**0.49**	**0.63**	-0.07	**0.53**	**0.65**
	*palA*	**0.63**	**0.57**	**0.57**	-0.08	**0.59**				**0.69**	**0.75**	**0.68**	**0.76**	-0.19	**0.46**	**0.55**
	*tolA*	*-0.52*	*-0.51*	-0.39	0.23	-0.31				-0.41	*-0.64*	-0.41	*-0.47*	0.04	*-0.67*	*-0.58*
Iron acquisition	*afuB*						**0.53**	**0.62**	-0.41							
	*ompP4*						**0.66**	**0.66**	-0.08							
	*hgbA*						0.39	0.16	-0.05							
	*apxIIA*									**0.57**	**0.83**	**0.60**	**0.80**	-0.34	**0.56**	**0.80**
	*comEA*									**0.57**	**0.86**	**0.60**	**0.65**	-0.09	**0.81**	**0.71**

n = 22, *P* < 0.05. Bold numbers: significant positive correlation; italicized numbers: significant negative correlation

**Table 3:** **Protein concentration ± SEM in lung tissue samples of controls and infected pigs**

**Protein (pg/ml)**	**Control**	**6 h p.i.**	***P*** **value** ^**2**^	**12 h p.i.**	***P*** **value** ^**2**^	**24 h p.i.**	***P*** **value** ^**2**^	**48 h p.i.**	***P*** **value** ^**2**^	**LOD (pg/ml)**
IL-1β	54 ± NA^1^	500 ± 64	<1.00E-08	304 ± 196	<1.00E-08	1037 ± 363	<1.00E-08	1322 ± 120	<1.00E-08	62.5
IL-6	113 ± 10	9419 ± 2015	4.06E-03	4423 ± 2165	0.07	1951 ± 501	2.88E-03	1975 ± 443	0.05	62.5
IL-8	82 ± 36	3544 ± 720	<1.00E-08	2386 ± 816	<1.00E-08	2181 ± 442	<1.00E-08	4416 ± 1114	<1.00E-08	62.5
IFNγ	15 ± 3	70 ± 12	4.06E-03	27 ± 14	0.45	19 ± 7	0.50	55 ± 21	0.16	6.2

^1^Only a single control sample was above limit of detection (LOD).

^2^ *P* values (control vs. time group) are calculated using Mann–Whitney *U* test.

## Discussion

Time course gene expression analysis provides valuable insight into the dynamics of the interdependent regulation of gene expression in host and pathogen during infection. However, the biological validity of the interactions inferred from transcriptional analysis depends on the comparability of gene expression results from the organisms involved. Reliable transcriptional profiles of both organisms are important in order to interpret the interdependent networks of gene expression. It is therefore ideal to simultaneously analyze the concurrent gene expression of both host and pathogen in the same biological sample. This should include using the same methods of RNA extraction, reverse transcription of RNA into cDNA, and qPCR, thereby excluding technical variation due the application of organism-specific methods.

In this study, the concurrent expression of selected host and pathogen genes was analyzed simultaneously in lung tissue sampled during the first 48 hours of *A. pleuropneumoniae* infection in pigs*.* The histopathological findings correlate with lesions reported in pigs experimentally infected with *A. pleuropneumoniae* in other studies [49,50]. The variations in severity and dissemination of lung lesions among pigs, especially at later time points, reflect the progressive nature of *A. pleuropneumoniae* infections with continuous development of new lesions. PCA of all bacterial gene expression data identified well defined groups of animals according to time of sampling. Less variation was seen among animals at 6 h p.i. compared to later time points, reflecting the disease progression which was also demonstrated in the histopathological findings.

TLR4 is an important part of the host defense against Gram-negative bacteria. It recognizes bacterial LPS with the help of co-receptor CD14 and the auxiliary proteins LBP and MD2 [51]. Except for *LBP*, expression of these TLR4-related genes were found to correlate positively and significantly with expression of bacterial genes involved in cell wall biogenesis and synthesis of LPS. These included LPS biosynthesis protein *kdsB* [52] and O-antigen translocase *wzxE* [53], as well as outer membrane lipoprotein/O-antigen processing protein *palA* [54], enterobacterial common antigen (ECA) biosynthesis protein *wecC*, and murein transglycosylase C *mltC* involved in synthesis of peptidoglycan and lipoprotein. The porcine innate immune system in the lung responds to the presence of *A. pleuropneumoniae* by a rapid and transient increase in expression of *TLR4* and co-factors. These genes related to the first line of defense peak within the first 6 h of infection and remain significantly up-regulated throughout the initial 48 h of infection. Even though TLR4 is an essential factor in the innate immune response to Gram-negative bacterial infections, expression of this PRR in response to *A. pleuropneumoniae* infection has not previously been extensively characterized. Here, we report the first characterisation of pulmonary expression of *TLR4* and co-receptors in response to *A. pleuropneumoniae* infection.

As indicated by high correlations of expression, the majority of bacterial pathogen associated molecular patterns (PAMPs) and cell wall biogenesis related genes investigated in this study are regulated in parallel with porcine PRR genes within the first 48 h of bacterial lung infection. Even though TLR4 and its ligand LPS have been widely studied [51,55,56], the present simultaneous transcriptional analysis of host and pathogen provide novel insight into the time course of PAMP synthesis and PRR-dependent inflammatory response to *A. pleuropneumoniae* at the site of infection. These important players could be fundamental in novel immune modulation approaches to improve host response to vaccination or antibiotic therapy.

*In vivo* and *in vitro* experiments have identified bacterial cell envelope biogenesis and maintenance category of genes as highly affected during infection [28,30,32]. Here we demonstrate the importance of these processes in the early stage of infection, as bacterial cell wall associated genes such as *kdsB*, *wecC,* and *wzxE* were all found to be most highly expressed within the initial 6 h. This conclusion is supported by cDNA microarray results from a large-scale study using the same biological material [23]. Lipoprotein encoded by *ompA* has been attributed with functions such as adherence to respiratory mucosal surfaces and maintenance of cell structural integrity in Gram-negative bacteria [57] and has previously been found important for the virulence of *A. pleuropneumoniae* [26]. In the present study, *ompA* expression was negatively correlated with the extensive expression of proinflammatory cytokines, and might be of less importance in induction of the substantial but transient inflammatory response to *A. pleuropneumoniae.* However, *ompA* was found to correlate positively and significantly with *SAA* expression. Human SAA has been reported to function as an opsonin for several Gram-negative bacteria, by binding to *ompA* [58]. As reported here, porcine *SAA* and bacterial *ompA* exhibit expression patterns that would allow a similar opsonisation mechanism in porcine pleuropneumonia.

A proinflammatory cytokine response, with high gene expression and tissue protein levels of IL-1, IL-6, and IL-8, was also observed locally in the porcine lung. Expression of these three genes was also previously found to be up-regulated in the liver compared to levels in control animals [14]. Thus, PRR-mediated pulmonary recognition of pathogen leads to a rapidly disseminated cytokine and APP response in the liver. Here, we also observed a local APP response at the site of infection, namely differential expression of *SAA*, *HP*, and *TF*. Pulmonary regulation was generally at lower (*SAA* and *TF*) or similar (*HP*) levels compared to the hepatic response reported in [14]. Pulmonary SAA expression appeared to be delayed compared to hepatic expression; we observed SAA expression levels in the lung at 24–48 h p.i. that were comparable to hepatic levels at 14–18 h p.i. reported in [14]. The importance of pulmonary induced *HP* is evident from its role in binding and sequestering hemoglobin as an iron source for invading bacteria under hemolytic conditions. The hemolytic activity of Apx-toxins, e.g. *apxIIA*, makes this highly relevant during *A. pleuropneumoniae* infection. Down-regulation of *TF* in order to prevent transport of iron from external sources to the site of infection might be another host strategy to lower the level of iron available to the invading bacteria. Accordingly, both pulmonary and hepatic [14] *TF* expression is decreased during infection.

The ability to cope with iron-restricted conditions in the host environment during infection is an important virulence factor for bacteria [59], and *A. pleuropneumoniae* has developed several strategies to overcome this challenge [8,21]. Hemoglobin binding protein *hgbA* has been reported to be solely responsible for uptake of hemoglobin in *A. pleuropneumoniae* [60], yet no differential expression of this gene was observed during infection in the present study. This is in agreement with microarray analyses reported in [23], where neither differential nor high expression of well-characterized iron-acquisition genes such as *hgbA* and *tbpBA* was observed from 6 h to 48 h p.i. in the same lung material as applied here. In contrast, we found expression of the genes *afuB* and *ompP4* to be differentially expressed from 6 h to 48 h p.i. with the highest observed levels occurring at 6 h p.i. *afuB* encodes a ferric uptake protein, and *ompP4* encodes a lipoprotein reported to be involved in acquisition of heme in *H. influenzae* [61]. The concurrent analysis and significant correlations between expression of the porcine *HP* and *TF* and bacterial *afuB* and *ompP4* peaking short time after bacterial colonization, reveals urgency for both organisms in the attempt of controlling the available iron at the site of infection. The lack of *hgbA* regulation and the decreasing expression of the iron acquisition genes *afuB* and *ompP4* over the course of infection may be evidence of iron acquisition becoming less challenging for the bacteria as the infection progresses. This could be due to the continuous development of hemorrhagic lesions, leading to hemoglobin becoming a more easily available source of iron.

The ability of *A. pleuropneumoniae* to adhere to host cells and secrete Apx-toxins right at the cell surface contribute to the establishment of hemorrhagic lesions and to the induction of a local immune response in the lung [21]. Two of the proteins that facilitate these functions are encoded by the genes *comEA* and *apxIIA* [38,62]. These were the two most highly regulated bacterial genes in the present study. As necrosis of bronchial and bronchiolar epithelium was established and progressed, we observed a gradual decrease of *apxIIA* and *comEA* expression towards the lowest observed expression at 48 h p.i. This result is in contrast to previous microarray results, where *ApxIIA* was neither significantly differentially expressed nor constantly highly expressed [23]. In the same study *mltC*, *palA*, *ompA* and *tolA* were all constitutively highly expressed during the first 48 h post experimental challenge, whereas these four genes were found to be regulated during infection with *A. pleuropneumoniae* in the present study. This discrepancy may be due to the higher sensitivity of the RT-qPCR method compared to the microarray method used. The genes *csgG* and *dnaK*, which may also trigger a host immune response, were constitutively expressed in both studies. Bacterial genes involved in exotoxin production were recently studied after *in vitro* infection of alveolar macrophages by *A. pleuropneumoniae* [63]. Although several Apx-toxins were found to be up-regulated during the course of infection, *apxIIA* was significantly down-regulated, which is in disagreement with results obtained in the present study. This discrepancy is likely a result of the former *in vitro* setup compared to the present *in situ* analysis at the site of infection.

IL-18 is a known inducer of IFN-γ during bacterial infection [64], and results presented here demonstrate that these two cytokines are concurrently up-regulated within the first 6 h of *A. pleuropneumoniae* infection. After this time point, *IL18* expression continues to increase and remains elevated throughout the first 48 h p.i. However, *IFNG* expression decreases to levels close to those observed in uninfected control animals by 12 h p.i. The very transient up-regulation of *IFNG* is supported by ELISA determinations of IFN-γ protein concentrations. IFN-γ promotes NK-cell activity and Th-1 cell differentiation which might be of less importance during bacterial pleuropneumonia in pigs.

Protein and mRNA levels of IL-8 were found to be highly and significantly elevated in the lung tissue of all infected animals, being present during the whole time interval studied here. IL-8 is the major chemoattractant for neutrophils during pulmonary infections and has previously been reported to be up-regulated in lung tissue 14–18 h after infection with *A. pleuropneumoniae* [15]. Here we found both protein and mRNA levels of IL-8 to correlate with increased infiltration of neutrophils and mononuclear inflammatory cells. Expression of mRNA coding for *IL8* in microdissected tissue surrounding colonies of *A. pleuropneumoniae* was highly consistent with these findings. IL-17A is a well-known proinflammatory cytokine, and like IL-8, it is a mediator of neutrophil recruitment. IL-17 has previously been proposed to play a protective role during Gram-negative pulmonary infections in mice [65,66]. IL-17A is implicated in the induction of several of the cytokines found to be up regulated in the present study. Here we present the first report of *IL17* expression in response to *A. pleuropneumoniae*. Differential pulmonary expression of *IL1B, IL6, IL8, TNF*, and *SAA* in response to *A. pleuropneumoniae* has previously been reported [15,17,31,67]. However, this is the first study to confirm this response in the immediate surroundings of the bacterial colonies using laser capture microdissection.

This work has implications for future studies of interdependent host-pathogen gene expression. Our results demonstrate that both organisms can easily be analyzed simultaneously under the exact same experimental conditions. Our customizable, chip-based high-throughput qPCR platform was found to be ideal for the present hypothesis-driven transcriptional analysis of host-pathogen interactions. Alternatively, if it is possible to extract sufficient amounts of high quality RNA from all organisms involved, sequencing methods could be applied if a deliberate and informed selection of genes for analysis cannot be made, or if global transcriptional screening is more suitable to answer the research question [5].

## Conclusions

By innovative application of high-throughput RT-qPCR we demonstrate the feasibility and applicability of dual-organism transcriptional analysis, and provide new insights into the dynamic interactions of pig and bacteria during pleuropneumonia. Temporal resolution of histopathological changes and differences in mRNA and protein levels of key components within the innate immune system as well as bacterial virulence and survival mechanisms were demonstrated during the first 48 hours of *A. pleuropneumoniae* infection. By applying laser capture microdissection, porcine gene expression could be confirmed in the immediate surroundings of the invading pathogen. Transcriptional up-regulation of selected cytokines was found to be reflected in the regulation of the tissue protein levels of these cytokines, correlating with infection status and histopathological findings.

### Availability of supporting data

High-throughput qPCR data are available in Additional file 1: Table S1; the table includes raw C_q_ values, experimentally obtained PCR efficiencies, sample grouping information, and challenge serotype information for all samples and assays that were used to yield the data presented in the present study.

Additional file 2: Table S2 contains qPCR primer sequences, information regarding qPCR primer T_m_, amplicon length, and experimentally obtained PCR efficiencies.

Additional file 3: Figure S1 contains a PCA of all bacterial gene expression data.

## Additional files

Additional file 1: Table S1. Raw high-throughput RT-qPCR data. Additional file 2: Table S2. qPCR primers employed in the study. Additional file 3: Figure S1. Principle component analysis of bacterial gene expression profiles.
