# Supplementary material for: Concurrent host-pathogen gene expression in the lungs of pigs challenged with Actinobacillus pleuropneumoniae
Source: BMC Genomics. 2015 May 28;16(1):417. doi: 10.1186/s12864-015-1557-6 (PMC4446954; doi:10.1186/s12864-015-1557-6)
Supplement: Additional file 1: Table S1. — Raw high-throughput RT-qPCR data. [file 12864_2015_1557_MOESM1_ESM.pdf]

Supplemental Table S1 – qPCR primers employed in the study

Gene Symbol	Description, protein name	Forward sequence (5´-3´)	Tm (°C)	Reverse sequence (5´-3´)	Tm (°C)	Amplicon length (bp)	Reaction efficiency
ACTB	Actin, beta	CTACGTCGCCCTGGACTTC	61	GCAGCTCGTAGCTCTTCTCC	59	77	0.78
PPIA	Peptidylprolyl Isomerase A (Cyclophilin A)	CAAGACTGAGTGGTTGGATGG	61	TGTCCACAGTCAGCAATGGT	60	138	0.82
C3	Complement Component 3	ATCAAATCAGGCTCCGATGA	61	GGGCTTCTCTGCATTTGATG	61	76	0.92
CFB	Complement factor B	TGATGGAGCGGGGTACTG	61	TCGGCTGCAGTAGTAGGTGA	60	103	1.07
CFD	Complement factor D	CCTCGGAGCAGCTGTATGT	59	ATGCCATGTAGGGTCTCTCG	60	89	0.90
MASP2	Mannan-binding lectin serine peptidase 2	GGCAAGGACAGCTGTAAAGG	60	TTCCTCCCACAAACCACTTC	60	82	0.96
TLR2	Toll-like receptor 2	GTTTTACGGAAATTGTGAAACTG	58	TCCACATTACCGAGGGATTT	59	136	0.54
TLR4	Toll-like receptor 4	TTTCCACAAAAGTCGGAAGG	60	CAACTTCTGCAGGACGATGA	60	145	1.05
CD14	CD14 molecule	GGGTTCCTGCTCAGATTCTG	60	CCCACGACACATTACGGAGT	61	165	0.80
MYD88	Myeloid differentiation primary response 88	CCAGACTAAGTTTGCACTCAGC	59	AGGATGCTGGGGAACTCTTT	60	99	0.98
LBP	Lipopolysaccharide binding protein	CCCAAGGTCAATGATAAGTTGG	61	ATCTGGAGAACAGGGTCGTG	60	83	0.92
CD163	CD163 molecule	CACATGTGCCAACAAAATAAGAC	59	CACCACCTGAGCATCTTCAA	60	130	0.86
MD2	Lymphocyte antigen 96	CAGTAAAGGTTGAGCCCTGTG	60	TTTGCGCATTGGTAAAGTCA	60	140	0.94
IRF3	Interferon regulatory factor 3	GCTACACCCTCTGGTTCTGC	60	GAGACACATGGGGACAACCT	60	95	0.99
IL1B	Interleukin 1, beta	CCAAAGAGGGACATGGAGAA	60	GGGCTTTTGTTCTGCTTGAG	60	123	1.06
IL1RN	Interleukin 1 receptor antagonist	GTTGGACGCAGTTAACATCACA	61	GCTGACTCAAAGCTGGTGGT	60	105	1.02
IL6	Interleukin 6	TGGGTTCAATCAGGAGACCT	60	CAGCCTCGACATTTCCCTTA	60	116	0.88
IL8	Interleukin 8	GAAGAGAACTGAGAAGCAACAACA	60	TTGTGTTGGCATCTTTACTGAGA	60	99	0.85
IL17A	Interleukin 17A	TACTCCAAACGCTCCACCTC	60	AGCATTGATACAGCCCGAGT	60	111	0.97
IL18	Interleukin 18 (interferon-gamma-inducing factor)	CTGCTGAACCGGAAGACAAT	60	TCCGATTCCAGGTCTTCATC	60	110	0.91
TNF	Tumor necrosis factor	CACGTTGTAGCCAATGTCAAAG	61	GAGGTACAGCCCATCTGTCG	61	129	0.91
TNFAIP3	Tumor necrosis factor, alpha-induced protein 3	CCCAGCTTTCTCTCATGGAC	60	TTGGTTCTTCTGCCGTCTCT	60	113	0.92
SAA	Serum amyloid A	CAGAGATGGGCATCATTCCT	60	TGGCATCGCTGATCACTTTA	60	184	0.96
HP	Haptoglobin	ACAGATGCCACAGATGACAGC	61	CGTGCGCAGTTTGTAGTAGG	60	105	0.95
TF	Transferrin	CTCAACCTCAAAACTCCTGGAA	61	CCGTCTCCATCAGGTGGTA	59	82	1.01
LTF	Lactotransferrin	GGAAAAGACTGCCCAGACAA	60	ACACTCCGTGTTGTCGTTGA	60	78	1.00
IFNg	Interferon, gamma	CCATTCAAAGGAGCATGGAT	60	TTCAGTTTCCCAGAGCTACCA	60	76	0.89
GZMB	Granzyme B	CCAGGACCAGGATAATCGAA	60	GGGTGACGTTGATTGAGCTT	60	101	1.04
CSF2	Colony stimulating factor 2	CCGAGGAAACTTCCTGTGAA	60	GCAGTCAAAGGGGATGGTAA	60	92	1.08
SFTPA	Surfactant protein A	CATGGGTGTCCTCAGTTTCC	60	CATCAAAAGCGACTGACTGC	60	86	0.91
SFTPD	Surfactant protein D	AGCGGAGCAGAGAACTGTGT	60	CTCAGAACTCGCAGATCACG	60	94	0.91
kdsB	3-deoxy-manno-octulosonate cytidylyltransferase	GGAACGTTTAGCGGAAGTGA	60	CAACGGCTCATCACCTTGTA	60	82	1.06
wzxE	Lipopolysaccharide biosynthesis protein	GCGCGATTTACTGATGGAAT	60	AAAACCGCCGTAATCAGTTG	60	102	0.96
comEA	DNA uptake protein	CCTAATAATCCGCCCGTTTC	61	CGGCAGTTTTGTTCGTTGT	60	70	0.99

hgbA	Hemoglobin-binding protein A	ATAAGGTCTTGCCGTTGCAC	59	AGACGAAAGAAGCCGCATTA	59	155	0.76
ompP4	Lipoprotein E	TGCGCAAGTGCTTGATATTC	58	GCTCATCACGATACGAGCAA	58	104	0.91
apxIIA	Hemolysin A (RTX-II toxin determinant A)	GAATCATTTCCATCGCCTGT	58	TCGATGGAGGAAACGGTAAC	58	112	0.92
mltC	Membrane-bound lytic murein transglycosylase C	AAGATGGCAAAACGTTACGG	60	TTCGCATAACTGACCGCATA	60	95	0.95
dnaK	Chaperone protein DnaK	ATCCTCGTTGGTGGTCAAAC	60	CTTCATCCGGATTCACGTCT	60	97	0.90
afuB	Ferric transport system permease protein fbpB	GTTATTACGTCCGGCATTGG	60	ACGTCAAAGCTACCGCCTAA	60	99	0.99
mgsA	Methylglyoxal synthase	CCGGATGTCAAAGCGTTAAT	60	GAAATTGGCACTGGTTTCGT	60	78	0.93
wecC	UDP-N-acetyl-D-mannosamine dehydrogenase	CGCACGAGAAGTAAACGACA	60	AAACAATTTCGGCGATTCAC	60	90	0.97
csgG	Putative lipoprotein	GACGAGGCAAAGAGCAAGTC	60	CCTTGCACCGAATAAACCAC	60	88	0.93
tolA	Cell envelope integrity inner membrane protein TolA	GCAAGCGGAAGAAGCTACAC	60	TTTTGCCGCAGCTTCTAAAT	60	91	0.89
palA	Outer membrane protein PalA	CGTGCAGACGCAGTTAAAAA	60	CCGGTTTTTCTTCACCGTAA	60	82	0.84
ompA	Outer membrane protein P5	TTGGGTGCAGGTGTTGAATA	60	TCTTGTGTGCCTGAACGAAC	60	116	0.76
csrA	Carbon storage regulator homolog	CATCATCCGCTAATGCTTTG	58	ATTACCGTTCTCAGCGTTCG	59	115	0.74
manB	Phosphomannomutase	AGAAGAAGCAACGGTCGAAA	59	ACCCGATTCTGCATGAAAAC	58	105	0.88
