# Supplementary material for: Concurrent host-pathogen gene expression in the lungs of pigs challenged with Actinobacillus pleuropneumoniae
Source: BMC Genomics. 2015 May 28;16(1):417. doi: 10.1186/s12864-015-1557-6 (PMC4446954; doi:10.1186/s12864-015-1557-6)
Supplement: Additional file 3: Figure S1. — Principle component analysis of bacterial gene expression profiles. [file 12864_2015_1557_MOESM3_ESM.pdf]

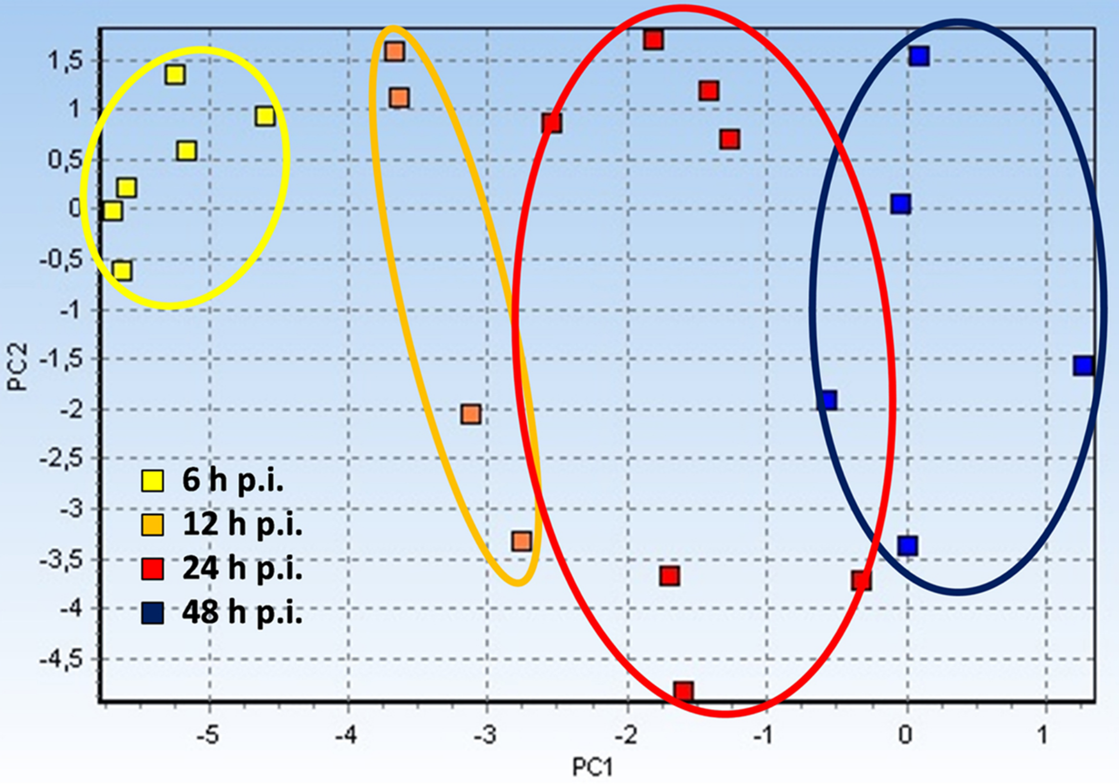

Supplementary Figure S1. Principal component analysis of bacterial gene expression profiles. Each point represents an individual sample.
